# Prevalence of *Plasmodium falciparum* histidine rich protein 2/3 gene deletions affecting rapid diagnostic test performance in travellers

**DOI:** 10.1093/jtm/taag007

**Published:** 2026-02-26

**Authors:** Olivia Detzlhofer, Felix Lötsch, Jessica Kirchner, Renate Schneider, Matthias Weiss-Tessbach, Stefan Winkler, Julia Walochnik, Lorenz Schubert

**Affiliations:** Department of Medicine I, Clinical Division of Infectious Diseases and Tropical Medicine, Medical University Vienna, Waehringer Guertel 18-20, 1090 Vienna, Austria; Division of Clinical Microbiology, Clinical Institute of Laboratory Medicine, Medical University of Vienna, Waehringer Guertel 18-20, 1090 Vienna, Austria; Department of Medicine I, Clinical Division of Infectious Diseases and Tropical Medicine, Medical University Vienna, Waehringer Guertel 18-20, 1090 Vienna, Austria; Institute of Specific Prophylaxis and Tropical Medicine, Medical University of Vienna, Kinderspitalgasse 15, 1090 Vienna, Austria; Department of Clinical Pharmacology, Medical University of Vienna, Waehringer Guertel 18-20, 1090 Vienna, Austria; Department of Medicine I, Clinical Division of Infectious Diseases and Tropical Medicine, Medical University Vienna, Waehringer Guertel 18-20, 1090 Vienna, Austria; Institute of Specific Prophylaxis and Tropical Medicine, Medical University of Vienna, Kinderspitalgasse 15, 1090 Vienna, Austria; Department of Medicine I, Clinical Division of Infectious Diseases and Tropical Medicine, Medical University Vienna, Waehringer Guertel 18-20, 1090 Vienna, Austria

**Keywords:** Malaria, *Plasmodium falciparum*pfhrp2/3 gene deletion, rapid diagnostic test, histidine-rich protein, diagnostic failure, travel medicine

## Abstract

In this traveller cohort, *pfhrp2/3* deletions occurred in 4 of 115 (3.5%) patients, with no double deletions observed. Notably, 51.3% of patients had visited countries anticipated to surpass the WHO 5% *pfhrp2/*3 threshold by 2028. Continued monitoring of *pfhrp2/3* deletions in non-endemic settings is needed to guide testing strategies.

Malaria remains a considerable risk for international travellers. In 2022, a total of 6131 malaria cases were reported across the European Union and European Economic Area.^1^ Timely diagnosis is critical to prevent severe disease and complications, underscoring the need to maintain proficiency in malaria diagnostics.^2^

Light microscopy is the diagnostic gold standard, as it allows prompt diagnosis, species identification and estimation of parasite density, which serves as a proxy for disease severity. However, its reliability is dependent on well-trained laboratory personnel, which has been a reason for the widespread implementation of RDTs as complementary diagnostic tools.^3^ The histidine rich protein (HRP) 2, an antigen produced by the parasite, is the primary target for *Plasmodium falciparum*-specific RDTs. *Plasmodium falciparum* also expresses HRP3, which can compensate for the absence of HRP2 in HRP2-based RDTs.^4^ Deletions of *pfhrp2* and *pfhrp3* (*pfhrp2/3*) causing false negative RDTs, have been challenging malaria control strategies in numerous African countries. The World Health Organisation (WHO) has published guidelines to monitor *pfhrp2/3* gene deletions, and recommended an adaptation of national testing strategies if ≥5% of symptomatic *P. falciparum* infections are missed by HRP2-based RDTs. African countries already exceeding the WHO threshold of 5% are Djibouti, Eritrea, Ethiopia, Sudan, South Sudan and Equatorial Guinea, whereas several other countries are expected to reach the 5% threshold by 2028.^5^ The increasing prevalence of *pfhrp2/3* deletions is likely to affect healthcare providers and laboratory personnel in non-endemic settings, however their implication for diagnostic strategies is less well defined.^6^ Accordingly, the objective of this study was to describe the prevalence of *pfhrp2/3* gene deletions and assess their potential impact on malaria diagnostic strategies at the Austrian National Reference Laboratory.

This retrospective laboratory-based observational study was conducted to describe the prevalence of *pfhrp2/3* gene deletions in all *P. falciparum*-positive samples referred to the Austrian national reference laboratory during 2019–2024. The Austrian national reference laboratory receives the majority of cases from Eastern Austria, in addition to a proportion of cases from other regions.^1^ Baseline demographics, and information on the disease and treatment were obtained from the electronic patient information system. Detailed descriptions of the PCR and qPCR protocols, as well as the statistical analysis methods, are provided in the Supplementary material.^7^^,^^8^ The study protocol was approved by the Ethics Committee of the Medical University of Vienna, Austria (ECS 1150/2022) and all study-related procedures were conducted in accordance with the Declaration of Helsinki. This study was conducted and reported in accordance with the STROBE guidelines.

Overall, 115 patients were included into the analysis. All patients for whom the travel history was known had travelled to Africa (Table 1). Only one (0.9%) patient had been travelling to an already described *pfhrp2/3* high risk country, namely South Sudan. However, 59 (51.3%) patients had travelled to a country which is suspected to reach the 5% threshold by 2028.^5^

**Table 1:** Countries of travel of the patients

Countries of travel	*n* (%)
Nigeria	33 (28.7)
Uganda	9 (7.8)
Cameroon	6 (5.2)
Kenya	5 (4.3)
Tanzania	5 (4.3)
Ghana	3 (2.6)
More than one country^a^	4 (3.5)
Other^b^	14 (12.2)
Not known	36 (31.3)
Overall	115

a Ghana and Togo (*n* = 1, 0.9%); Kenya and Tanzania (*n* = 1, 0.9%); Kenya, Tanzania and Uganda (*n* = 1, 0.9%), Senegal, Gambia, Guinea-Bissau and Sierra Leone (*n* = 1, 0.9%)

b Angola (*n* = 1, 0.9%), Benin (*n* = 1, 0.9%), Burkina Faso (*n* = 1, 0.9%), Côte d’Ivoire (*n* = 2, 0.9%), Gabon (*n* = 1, 0.9%), Democratic Republic of the Congo (*n* = 2, 1.7%), Madagascar (*n* = 1, 0.9%), Niger (*n* = 1, 0.9%), Zambia (*n* = 1, 0.9%), Zanzibar (*n* = 1, 0.9%), Senegal (*n* = 1, 0.9%) and South Sudan (*n* = 1, 0.9%). Bold listed countries are currently listed as high-risk country; underlined countries are suspected to reach the 5% threshold by 2028.^5^

The median age of the cohort was 39.2 (25.2–49.5) years, and only 32.5% (25 of 115) were female. In 46 of 115 patients (40%) information on disease severity was available, 12 of these (26.1%) experiencing severe malaria. Treatment regimens for patients with severe malaria comprised artesunate (*n* = 5, 41.7%), artesunate and clindamycin (*n* = 2, 16.7%), quinine and doxycycline (*n* = 1, 8.3%), artemether/lumefantrine (*n* = 3, %) and not reported (*n* = 1, 8.3%). Uncomplicated malaria had been treated with artemether/lumefantrine in all cases.

The analyses revealed four *pfhrp2* or *3* gene deletions (4 of 115, 3.5%), but no case of deletions of both genes in one same sample. *Pfhrp2* gene deletions were detected in a patient from Ghana and in a patient from Uganda. The *pfhrp3* gene deletions were found in a patient who had travelled to Kenya and Tanzania and in a patient who had travelled to Senegal. Figure 1 gives an overview of countries travelled by patients treated for malaria at our centre as well as their *pfhrp2/3* gene deletion status. Characteristics of patients with *pfhrp2/3*-deleted *P. falciparum* malaria are given in Supplementary Table 1.

**Figure 1:**
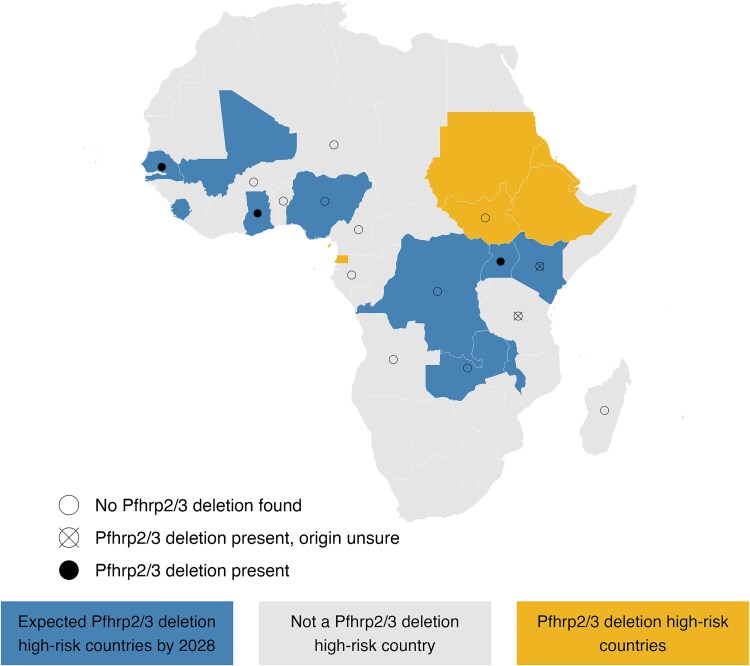
Map showing African countries visited by malaria patients in this study, stratified by their Pfhrp2/3 gene-deletion risk status, alongside the Pfhrp2/3 deletion status of the detected infections

The emergence of *pfhrp2/3* gene deletions has challenged diagnostic strategies in countries primarily reliant on HRP2-based RDTs for *P. falciparum* infection. While the impact of these deletions in endemic settings has been increasingly described, their implications for diagnostic strategies in non-endemic centres remain less well defined. In general, the WHO recommends an adaptation of diagnostic strategy for regions where ≥5% of symptomatic *P. falciparum* infection are missed by HRP2-based RDTs. In this study, comprising all malaria tropica patients diagnosed at the Austrian national reference laboratory over the past 5 years, *pfhrp2/3* deletion was 3.5%, remaining below the WHO threshold. However, more than half of the travellers (52.8%) had returned from countries considered at high risk of reaching this threshold by 2028.

Alternative testing strategies include light microscopy, PCR or alternative non-HRP based RDTs. Light microscopy, the current gold standard, allows prompt diagnosis and species identification, while not needing complex equipment. However, accurate interpretation depends on the availability of experienced microscopists, which may be challenging in smaller hospitals that encounter malaria cases infrequently. In such contexts, PCR-based methods might be a suitable alternative.^9^ Additionally, new RDT combinations might be of value. A recent analysis of RDTs in a high-risk setting combining HRP2 and *PfLDH* in a single test line, demonstrated better test results (77%) in comparison to HRP2-based RDTs (55.9%), however a significant number of infections is still missed.^10^

The main limitation of this study is its single-centre design, which reflects the prevalence of *pfhrp2/3* gene deletions observed at the Austrian national reference laboratory. Consequently, the prevalence of *pfhrp2/3* gene deletions may differ in centres caring for patient populations with more frequent travel to regions at high risk for *pfhrp2/3* gene deletions.

In conclusion, the overall prevalence of *pfhrp2/3* gene deletions among travellers over the past five years remained low. Given the expected rise in *pfhrp2/3* deletions in several African countries, non-endemic reference centres should continue monitoring for *pfhrp2/3* deletions to guide national diagnostic testing strategies.

## Supplementary Material

Supplementary_file_pfhrp_travellers_research_letter_revision_taag007

## Data Availability

Data available on request.
