# Supplementary material for: Prevalence of Plasmodium falciparum histidine rich protein 2/3 gene deletions affecting rapid diagnostic test performance in travellers
Source: J Travel Med. 2026 Feb 26;33(3):taag007. doi: 10.1093/jtm/taag007 (PMC13042232; doi:10.1093/jtm/taag007)
Supplement: Supplementary_file_pfhrp_travellers_research_letter_revision_taag007 [file supplementary_file_pfhrp_travellers_research_letter_revision_taag007.docx]

**Supplementary file: Prevalence of Plasmodium falciparum histidine rich protein 2/3 gene deletions affecting rapid diagnostic test performance in travellers**

**Supplementary methods and statistical analysis**

Supplementary methods

At our centre light microscopy remains the primary diagnostic method, with PCR used to confirm cases of high clinical suspicion despite negative microscopy or to clarify potential co-infections. No RDTs were routinely performed. PCR were performed in all *Plasmodium falciparum* positive samples, which are routinely stored at the Austrian national reference laboratory. The probe-based quantitative real-time PCR (4plex) was performed following the protocol published by Kreidenweiss et al..^1^ Final concentrations of the qPCR mix were 1× HOT FIREPol® Multiplex Master Mix (Solis Biodyne), 400 nM each for *pfhrp2* and *pfhrp3* primers, 600 nM for pfbtub primers, and probe concentrations of 300 nM (pfbtub), 75 nM (*pfhrp2*), and 150 nM (*pfhrp3*). Reactions were performed in 20 µl containing 2 µL template DNA and were run on the QuantStudioTM 5 Real-Time PCR System (ThermoFisher Scientific). Final thermal cycling conditions were set to an initial activation at 95°C for 10 min, followed by 45 cycles of 95°C for 20 sec to 60°C for 1 min at a ramp rate of 1.6°C/s. Samples were assayed in duplicate, with one positive and negative control per plate. HRP2-RDT test failures associated with *pfhrp2/3* gene deletions could not be assessed, as only stored DNA extracts were available.

Statistical analysis

Baseline characteristics are presented as medians with quartiles (Q1 – Q3) for continuous variables, and as counts with corresponding percentages for categorical variables. Data collection was supported by Microsoft Excel (Version 16.97). All statistical analyses and data visualizations was conducted using the R software (Version 2025.05.1+513.pro3).^2-4^

Supplementary table 1. Characteristics of patients with *pfhrp2/3*-deleted Plasmodium falciparum malaria.

| Patients | Demographics | Time to diagnosis (days) | Deletion | Month and Year of infection | Country of travel | CT-value | Severe  malaria |
| --- | --- | --- | --- | --- | --- | --- | --- |
| Patient 1 | 18, male | NA | *pfhrp2* | December 2023 | Ghana | 27 | no |
| Patient 2 | 26, female | 0 | *pfhrp2* | June 2019 | Uganda | NA | yes |
| Patient 3 | 65, male | NA | *pfhrp3* | February 2024 | Senegal | 9 | no |
| Patient 4 | 64, female | 5 | *pfhrp3* | February 2022 | Kenia and Tanzania | 8 | yes |

Abbreviations: NA, not applicable; *pfhrp*, *Plasmodium falciparum histidine-rich protein*

References:

1. Kreidenweiss A, Trauner F, Rodi M, et al. Monitoring the threatened utility of malaria rapid diagnostic tests by novel high-throughput detection of Plasmodium falciparum hrp2 and hrp3 deletions: A cross-sectional, diagnostic accuracy study. *EBioMedicine* 2019; **50**: 14-22.

2. Pebesma E. Simple Features for R: Standardized Support for Spatial Vector Data. *The R Journal* 2018; **10**(1): 439-46.

3. Wickham H. ggplot2: elegant graphics for data analysis. New York: Springer; 2009.

4. South A. rnaturalearth: world map data from Natural Earth [software]. 2017.
